# Analysis of randomised trials with long-term follow-up

**DOI:** 10.1186/s12874-018-0499-5

**Published:** 2018-05-29

**Authors:** Robert D. Herbert, Jessica Kasza, Kari Bø

**Affiliations:** 1 Neuroscience Research Australia (NeuRA), Sydney, Australia; 2 The University of New South Wales, Sydney, Australia; 3 Monash University, Melbourne, Australia; 4 Norwegian School of Sport Sciences, Oslo, Norway; 5 Akershus University Hospital, Lørenskog, Norway

**Keywords:** Clinical trials, Randomized controlled trials, Long-term follow-up, Non-compliance, Treatment switching, Co-intervention, Loss to follow-up

## Abstract

Randomised trials with long-term follow-up can provide estimates of the long-term effects of health interventions. However, analysis of long-term outcomes in randomised trials may be complicated by problems with the administration of treatment such as non-adherence, treatment switching and co-intervention, and problems obtaining outcome measurements arising from loss to follow-up and death of participants. Methods for dealing with these issues that involve conditioning on post-randomisation variables are unsatisfactory because they may involve the comparison of non-exchangeable groups and generate estimates that do not have a valid causal interpretation. We describe approaches to analysis that potentially provide estimates of causal effects when such issues arise. Brief descriptions are provided of the use of instrumental variable and propensity score methods in trials with imperfect adherence, marginal structural models and g-estimation in trials with treatment switching, mixed longitudinal models and multiple imputation in trials with loss to follow-up, and a sensitivity analysis that can be used when trial follow-up is truncated by death or other events. Clinical trialists might consider these methods both at the design and analysis stages of randomised trials with long-term follow-up.

## Background

Sometimes we want to know about the long-term effects of a health intervention. That is, we want to know the effects of an intervention long after it was first administered. Long-term effects of interventions for slowly progressing diseases and chronic conditions are of particular interest.

It is generally considered that the best way to determine the effects of a health intervention is to conduct a randomised trial. That implies the best way to determine the long-term effects of a health intervention is to conduct a randomised trial with long-term follow-up. An example of a randomised trial with an exceptionally long-term follow-up is the trial conducted by Fisher and colleagues that followed up patients with breast cancer 20 years after randomisation to mastectomy, lumpectomy, or lumpectomy and radiotherapy [1]. Another example is a trial conducted by Bø and colleagues that followed up women with stress urinary incontinence 15 years after randomisation to 6 months of home exercise or intensive pelvic floor muscle exercise [2].

This paper considers issues pertaining to the analysis of trials with long-term follow-up. The issues considered here also arise in randomised trials with short-term follow-up but are particularly problematic in trials with long-term follow-up. While the focus of this paper is on analysis of long-term efficacy and effectiveness, much of the discussion is also relevant to long-term safety and health economic analyses. For simplicity, we focus on trials in which participants are randomised to receive a treatment or control condition but much of the discussion applies equally to other randomised trial designs.

The paper begins by clarifying some terminology. Subsequently, consideration is given to problems that arise with the administration of treatment in long-term trials such as non-adherence, treatment switching and co-intervention, as well as problems in obtaining outcome measurements arising from loss to follow-up and death. A brief description is given of methods that might be used to obtain estimates of causal effects of intervention when such issues arise, and references are provided to key papers for further reading around each method. A summary is given in Table 1. No attempt is made to provide a comprehensive review of all available methods or a detailed explanation of any particular method. The paper concludes with some additional comments about the analysis of randomised trials with long-term follow-up.

**Table 1:** Summary. The table identifies some problems that arise in randomised trials with long-term follow-up, whether the problem affects acute, intermittent or sustained interventions; the estimand of primary interest; the primary methods used to obtain those estimands; and whether the analysis is applicable to cross-sectional analyses or longitudinal analyses

Problem	Does the problem affect acute, intermittent or sustained interventions?	What causal effects can be estimated?	Methods	Methods suitable for cross-sectional or longitudinal analyses?
Non-compliance	most commonly affects sustained or intermittent interventions, but can also affect acute interventions	the complier average causal effect (i.e., the average effect in people who would adhere to whichever treatment they were assigned to)	instrumental variable regression, propensity score methods	when there is a summary measure of compliance across the entire treatment period, both methods can be applied to cross-sectional or longitudinal analyses
Treatment switching	affects intermittent or sustained interventions	the average effect in people who do not switch treatments (or the average effect of some other defined treatment pattern)	marginal structural models, g-estimation	can be applied to cross-sectional analyses, but are most valuable when analyses are longitudinal
Loss to follow-up	this is not a treatment-level issue: loss-to-follow-up can occur with any type of intervention	the average treatment effect	mixed longitudinal models, multiple imputation	mixed longitudinal models are applicable to longitudinal analyses, multiple imputation can be applied to cross-sectional or longitudinal analyses
Truncation by death and other events	this is not a treatment-level issue: truncation can occur with any type of intervention	the survivor average causal effect (i.e., the average effect in people who would have survived no matter which treatment they were allocated to)	sensitivity methods, propensity score-based methods	methods are most well-developed for cross-sectional analyses, extensions of propensity score-based methods are available for longitudinal analyses

### Explanation of terms

#### The causal effect of an intervention

The causal effect of a health intervention on a person is the difference between that person’s outcomes with and without that intervention. In practice the causal effect of intervention is unobservable because we cannot simultaneously observe the person’s outcomes both with and without the intervention. Therefore, at least theoretically, the causal effect of an intervention on a person is unknowable. This has been called the fundamental problem of causal inference [3].

Even though the causal effect of an intervention on an individual person is unknowable it is possible to estimate the average causal effect of an intervention on a particular population, provided key assumptions are valid. The average causal effect can be estimated with a “perfect” randomised trial (i.e., a trial in which there is perfect adherence to randomised treatment, perfect blinding, no loss to follow-up, etc.). In randomised trials, randomisation of participants to groups creates groups that are “exchangeable” at baseline: on average, these groups are expected to be representative of the same population. This implies that on average the randomised groups will be similar at baseline with regards to measured, unmeasured, unmeasurable and unknown baseline variables. During the course of the trial, the intervention of interest is administered to participants in one group but not the other. One group provides an estimate of the population’s mean outcome with the intervention and the other group provides an estimate of the same population’s mean outcome without the intervention. If the trial is, in fact, “perfect”, then the difference in mean outcomes provides an estimate of the average causal effect of the intervention in the whole population and, under these ideal conditions, coincides with the “intention-to-treat” effect [4]. However, “perfect” trials are rare, if they exist at all. The extent to which trials depart from this perfect ideal tends to increase with the duration of follow-up.

#### Acute, intermittent and sustained interventions

When considering long-term effects of interventions, it is useful to differentiate between three types of interventions. *Acute* interventions are intended to be administered just once over a short period of time. An example of an acute intervention is the administration of intravenous recombinant tissue plasminogen activator soon after ischaemic stroke. Another example is the insertion of Harrington rods for scoliosis - most surgical interventions are acute interventions. *Episodic* interventions are those which are intended to be administered repeatedly or intermittently. An example of an episodic intervention is the use of triptan for treatment of migraine. *Sustained* interventions are those which are intended to be administered continuously. Examples of sustained interventions are dietary and exercise programs for weight loss.

#### Clinical and non-clinical protocol violations

In clinical trials, and especially clinical trials with long-term follow-up, things never go exactly to plan. That is, the trial protocol is often violated.

Some protocol violations are manifestations of the complexity and unpredictability of clinical care. For example, a clinician might intend to provide a specific intervention to a particular patient but, for any of a myriad of reasons, the intended intervention might never be delivered. Or a clinician might intend to provide a specific intervention but subsequently decide to switch treatments, or provide a co-intervention that was not anticipated in the initial care plan. We call these events *clinical protocol violations*.

Other protocol violations can only occur in the context of a clinical trial. For example a trial protocol may dictate that outcomes should be measured 12 and 36 months after randomisation but the first set of outcomes for a particular trial participant might actually be measured at 13 months and the second set of outcome measurements might not be obtained at all. To the extent that measurement of outcomes and 12 and 36 months is not part of normal clinical care these protocol violations could only occur in the context of a clinical trial. We refer to this type of protocol violation as *non-clinical protocol violations*.

#### Explanatory and pragmatic trials

Protocol violations are more problematic when the objective of the trial is explanatory rather than pragmatic. *Explanatory trials* are designed to determine the effects (or efficacy) of receiving a specific intervention whereas *pragmatic trials* seek to determine the real-world effects (or effectiveness) of the intention to provide a specific intervention [5–7].

Clinical protocol violations are of little concern in pragmatic trials because the purpose of pragmatic trials is to estimate the effect of an intention to intervene rather than the effect of the intervention itself. Even in the presence of clinical protocol violations, the difference in the mean outcomes of participants randomised to intervention and control groups (the so-called intention to treat estimate) can provide an estimate of the effect of the intention to intervene. Clinical protocol violations are of more concern in explanatory trials because the purpose of explanatory trials is to estimate the effects the intervention itself. In the presence of clinical protocol violations the intention to treat estimate differs from the effect of intervention. Non-clinical protocol violations such as loss to follow-up are of concern in both pragmatic and explanatory trials because non-clinical protocol violations distort both explanatory and pragmatic estimates of the effects. Clinical protocol violations need concern only explanatory trialists but non-clinical protocol violations should concern both explanatory and pragmatic trialists.

## Main text

### Non-adherence and non-compliance

Randomisation to intervention and control groups implies an intention to administer the intervention to participants in the intervention group and an intention to administer the control condition to participants in the control group. In practice those intentions might not be realised: some participants allocated to the intervention group might never receive the intervention and some participants allocated to the control group might receive the intervention. The failure of a participant to fully receive the (intervention or control) condition to which they were allocated is called non-adherence. Non-adherence is more often seen in trials of episodic and sustained interventions than in trials of acute interventions.

Non-adherence may or may not complicate estimation of the causal effects, depending on the purpose of the trial. If the purpose of the trial is pragmatic – that is, if the purpose is to determine the effect of an intention to treat – then analysis and interpretation is straightforward. Analysis by intention to treat (i.e. by randomised group) provides an estimate of the causal effect of the intention to provide an intervention even if some trial participants do not receive the intended intervention. In contrast, if the purpose of the trial is explanatory – that is, if the purpose is to determine the effect of actually receiving the intervention – non-adherence complicates analysis and interpretation. When there is non-adherence, the intention to treat analysis does not generally provide an estimate of the average causal effect of actually receiving the intervention. As a consequence, explanatory trialists may report estimates of the “per protocol” effect, obtained by excluding non-adherent participants from the analysis, or estimates of the “as treated” effect, obtained by analysing the data from non-adherent participants as if those participants had been allocated to the other group. Estimation of these effects involves comparison of groups that are not exchangeable.

To see why, consider a hypothetical trial comparing the effect of an exercise intervention to a control intervention on the self-reported pain of people with knee osteoarthritis. Suppose that people with higher levels of depression are less likely to adhere to the exercise intervention than non-depressed people. Suppose also that we know that depressed people typically report higher levels of pain. If that were the case, the “as treated” analysis, in which non-adherent participants in the intervention group are assigned to the control group, would compare an intervention group that includes few depressed participants with a control group that contains more depressed participants. So the groups would not be exchangeable. Similarly, the “per protocol” analysis that excludes those participants who did not adhere to their assigned intervention would also compare an intervention group that includes few depressed participants with a control group that contains more depressed participants. With both the as treated and per protocol analyses, the groups contain different proportions of depressed participants, and depression affects outcomes, so the estimates of treatment effect will be biased. To the extent that per protocol and as treated analyses compare non-exchangeable groups they do not have a valid causal interpretation [8, 9]. Arguably such analyses should not be conducted [10].

More satisfactory approaches seek to estimate the average causal effect of intervention in the “latent” (unobservable) subpopulation of compliers. This effect is known as the complier average causal effect or CACE. A complier is a person who would fully adhere to the intervention condition if assigned to the intervention group *and* would fully adhere to the control condition if assigned to the control group. Participants in a trial are either allocated to the intervention group or the control group but not both, so even if a participant adheres to the allocated condition we cannot know that he or she would also have adhered had he or she been allocated to the other condition. We can observe non-adherence but we cannot always observe non-compliance. That is why we call compliance a “latent” variable.

Even though we cannot always identify compliant trial participants, it may be possible to obtain valid estimates of the CACE using an instrumental variable approach. The instrumental variable approach assumes that the groups consisting of participants allocated to the intervention and control conditions are exchangeable. This assumption is met in properly randomised trials. (So, in this case, we say that the “instrumental variable” is randomised group.) Identification of the CACE using an instrumental variable approach also requires that the intervention has no effect on people who were assigned to the intervention group but actually received the control condition, and that the intervention has no effect on people who were assigned to the control group but actually received the intervention. In other words, it is assumed that assignment to the intervention or control arm of the trial itself has no effect on the outcomes of non-compliers. This is known as “exclusion restriction”. The validity of this assumption will depend on what the intervention and control conditions are and how they are applied: for many individually randomised trials the exclusion restriction assumption is likely to be valid. Another assumption is that there are no “defiers”. Defiers are people who would receive the intervention if allocated to the control group and would not receive the intervention if allocated to the intervention group.

When these assumptions are satisfied, the CACE is just the intention to treat effect divided by the difference in the proportions of participants in the intervention and control groups who receive the intervention [11]. We could estimate the CACE in this way but in practice the CACE is more commonly estimated with instrumental variable regression [12]. Non-technical introductions to the instrumental variable approach and instrumental variable regression are provided by Stuart and colleagues [11] and Angrist [13].

An alternative approach to estimating the CACE uses propensity scores [14]. This approach is most straightforward in trials where it is not possible for participants in the control group to access the intervention, as commonly occurs in trials of novel interventions such as trials of new drugs or new surgical techniques or new therapies. In such trials, participants in the control group are always adherent because they cannot receive the intervention; only participants in the intervention group can be non-adherent. In the propensity score approach, a model to predict adherence is constructed using only data from participants in the intervention group, adjusting only for variables measured prior to randomisation. This approach is most useful when adherence is a binary variable, so logistic models can be used to predict adherence and the predicted logits can be converted into probabilities. Subsequently inverse probability weighting can be used to adjust the control group’s outcomes so that the distribution of control group participants’ outcomes approximates the distribution that would have been observed if the control group had consisted entirely of participants who would have adhered if allocated to the intervention group. The CACE is then simply the difference in the mean outcome of the adherent participants in the intervention group and the adjusted mean outcome of participants in the control group. Instead of invoking the exclusion restriction assumption, the principal score approach requires that, conditional on the covariates used to model adherence, outcomes that participants would attain under either the treatment or control condition are independent of compliance. It has been found that when this “principal ignorability” assumption is not valid (i.e. there are unmeasured confounders of the relationship between complier status and potential outcomes), generating a model of adherence in the intervention group that is highly predictive may help reduce bias in the estimation of the CACE [14].

Both the instrumental variable and propensity score approaches make assumptions that are untestable. Nonetheless, the assumptions are quite plausible in some trials. Moreover, the assumptions made by the two approaches are very different: the instrumental variable approach assumes exclusion restriction whereas the propensity score approach assumes principal ignorability. So it may be possible to cross-validate estimates of the CACE obtained with the instrumental variable and principal score approaches [14].

The preceding discussion implicitly assumed that adherence is a binary variable, but in some contexts that will be simplistic. Adherence, particularly adherence to complex episodic or sustained interventions, may be a complex, multi-dimensional, quantitative construct. For example, the extent of adherence to an exercise intervention depends on the period for which the participant exercises, the frequency and duration of exercise sessions, and the intensity and quality of exercise carried out within sessions. Methods for dealing with multi-dimensional, quantitative measures of adherence are available but they are less well developed than methods that treat adherence as a binary variable. Both instrumental variable regression and the propensity score approach can accommodate quantitative measures of adherence [15, 16].

A specific type of partial adherence is treatment switching. Treatment switching will be discussed separately below. There are other types of non-adherence too. The trial protocol might dictate that participants are allocated to a control group or to a group that receives a specific intervention, but it may be that some trial participants receive an altogether different intervention. These behaviours are discussed under the heading of co-interventions.

### Treatment switching

Treatment switching frequently arises in the analysis of randomised trials with long-term follow-up. Trial participants allocated to a control group may adhere to the control condition for a while but then seek the intervention. In some trials, particularly trials of sustained interventions, participants may switch the other way: from the intervention to the control condition. Or they may switch to a different treatment altogether. Treatment switching can be thought of as time-dependent treatment exposure. Alternatively, it can be thought of as time-dependent non-adherence: the participant who switches intervention adheres for some time and then becomes non-adherent.

In some trials of acute interventions, switching occurs only in one direction. Consider, for example, a trial that randomises patients with anterior cruciate ligament ruptures to receive either a knee reconstruction followed by exercise or exercise alone. A participant allocated to the exercise group might adhere to the exercise program for a while but subsequently switch interventions and have a knee reconstruction. But there is an asymmetry here: it would not be possible for a participant who was allocated to the knee reconstruction group and received a knee reconstruction to subsequently switch interventions. In these examples the switching is unidirectional but in other examples, particularly trials of episodic and sustained interventions, switching may be bidirectional. For example, participants in a trial that randomises patients with Achilles tendinopathy to receive either in-shoe orthoses or an exercise program could switch from exercise to orthoses or from orthoses to exercise. Having switched, participants could switch back, so there is the potential for multiple switches by individual participants.

Treatment switching is a type of clinical protocol variation. So, at least in theory, the intention to treat estimate has a meaningful interpretation even when treatment switching occurs. The intention to treat estimate provides an estimate of the effect of an initial intention to provide a specific intervention, even if subsequently that intention is not fully realised because people switch interventions. However, in the presence of treatment switching even the most pragmatic trialist might find the intention to treat estimate unsatisfying. Most trialists and clinicians will want to know the effect of intervention in people who do not switch interventions.

Just as with estimation of the CACE, naïve estimates of the effect of intervention in people who do not switch interventions could be obtained from per protocol analyses or as treated analyses. And, just as with estimation of the CACE, the estimates obtained using these approaches may not have a causal interpretation so are not recommended. Another approach would be to include treatment as a time-varying covariate in the outcome model but this approach relies on assumptions that are not likely to be valid [17]. An alternative and better approach is to estimate the average causal effect of intervention in the population that would not switch intervention whether allocated to the intervention or control condition.

One widely applicable approach is the use of marginal structural models with inverse probability of censoring weights, where patients are artificially censored at the time of a switch. This approach can be used in longitudinal trials where outcomes are measured on multiple occasions, as is often the case in trials with long-term follow-up. In the simplest scenarios, in which only participants from the control group switch interventions, the approach proceeds as follows [18]. First, outcome data from control group participants who switched interventions are censored at the time of switching so that, for any trial participant, the only data included in the analysis are those measured prior to the switch. Then a longitudinal model is constructed using data only from participants allocated to the control group. The model predicts non-switching behaviour up until the time of each follow-up. The predictors in the model could either be variables measured prior to randomisation or variables measured after randomisation such as the outcome measured at the preceding follow-up time. The model is usually a logistic model, so the logit can be converted into a probability (the predicted probability not to switch intervention up to that point in time). The outcomes of control group participants are weighted by the inverse of the time- and participant-specific predicted probability of not switching intervention, so outcomes obtained from participants who did not switch (and who therefore were not censored) but had a high predicted propensity to switch interventions up to that point in time are more heavily weighted. The aim of weighting is to make the control group participants at any follow-up time more exchangeable with intervention group participants. (Remember that the intervention group also includes participants who, had they been allocated to the control group, would have switched intervention. However, because these participants were allocated to the intervention group they did not switch interventions and their data were not censored.) The difference between the weighted mean outcome of participants in the control group and the mean outcome up until the time of switching of participants in the intervention group provides an estimate of the causal effect of intervention in people who do not switch interventions. The primary assumption of this approach is that, conditional on the predicted probability of not switching interventions, the treated and control groups are exchangeable.

Another approach to the estimation of the effect of intervention on time-to-event outcomes in people who do not switch interventions is to construct rank-preserving structural failure-time models using g-estimation. G-estimation methods are not restricted to time to event outcomes (see Toh and Hernan [19] for an application of g-estimation to continuous outcomes). When applied to time to event data, Kaplan-Meier survival curves are stratified by received intervention (rather than allocated intervention): participants contribute person-time and censoring to the control condition’s survival curve while receiving the control, and they contribute person-time and censoring to the intervention condition while receiving intervention. Participants who switch intervention contribute some person-time to the control condition and some to the intervention condition. It is assumed that the effect of intervention, expressed as an acceleration factor, is the same for all trial participants and independent of when intervention is received. The acceleration factor is the factor by which intervention extends untreated survival time. If, as is assumed, the acceleration factor is constant, it is possible to estimate the acceleration factor and reconstruct the survival curves that would have been observed had treatment switching not occurred.

Latimer and colleagues [18] and Toh and Hernan [19] provide gentle introductions to the use of both marginal structural models with inverse probability of censoring weights and rank-preserving structural failure-time models using g-estimation to estimate causal effects of intervention in the presence of treatment switching.

### Differential co-intervention

Many clinical trial protocols discourage patients and clinicians from receiving or implementing interventions other than the specific interventions under study for the duration of the trial. That aspiration is often not achieved. Trial participants often receive co-interventions that are not specified in trial protocols. The co-interventions might be well-established interventions for which there is strong evidence of effect. Alternatively, co-interventions might be highly experimental or completely untested, or they might have been shown to have little or no effect. The most common co-interventions are probably those that are never documented: changes in physical activity (rest or exercise), dietary changes, and the care and support provided by family and friends.

To the extent that co-interventions influence outcomes and are differentially administered to participants in the intervention and control arms of the trial, co-interventions potentially distort estimates of the effect of intervention. Of particular concern for the interpretation of clinical trials (at least for explanatory trialists) is that trial participants with poor short-term outcomes may be more likely to receive effective co-interventions than trial participants with good short-term outcomes. The effect would be to reduce the size of the conventional (intention to treat) estimate of the effect of intervention.

Pragmatic trialists need not be too troubled by co-intervention. From a pragmatic perspective, co-intervention can be considered to be part of the interventions that are being compared in the trial. Consider, for example, a trial of tympanostomy for children with recurrent middle ear infections. Children allocated to the control group might receive more paracetamol and more ice-cream than children given tympanostomy, even if paracetamol and ice-cream are not specified in the trial protocol. The pragmatist might still consider that the intention to treat estimate of the effects of tympanostomy provided by this trial is useful because the intention to treat estimate quantifies the effect of tympanostomy compared to the co-interventions that would normally be applied if a tympanostomy was not done. The explanatory trialist, who has a more specific question about the precise effect of tympanostomy compared to a rigidly defined control condition, will be less satisfied with the intention to treat estimate.

The issue of co-intervention is similar to the issue of treatment switching, discussed above. However, trial participants typically seek many different sorts of co-intervention and typically co-interventions are poorly documented. This makes it very difficult to estimate the causal effect of intervention that would have been observed had co-intervention not occurred. The issue of co-intervention, perhaps more than any other, makes it difficult to obtain robust explanatory estimates of the long-term causal effects of interventions.

### Loss to follow-up

Almost all trials experience loss to follow-up. Participants may lose interest in continued participation in the trial or they may find that ongoing participation is onerous or unpleasant, in which case they may sever contact with trial staff and become lost to follow-up. Another cause of loss to follow-up is death, but we defer consideration of loss to follow-up caused by death until the next section. The problem of loss to follow-up is inevitably greatest in trials with long-term follow up.

There are many ways to analyse data in the presence of significant loss to follow-up. The simplest is to conduct the analysis on the available data from followed-up participants. The adequacy of this approach depends on whether a cross-sectional or longitudinal approach is taken to the analysis of the clinical trial. Simple cross-sectional analyses conducted on the available data from followed-up participants in the clinical trial (i.e., separate analyses conducted on the data obtained at each follow-up occasion) are potentially problematic. This is because the characteristics of intervention group participants lost to follow-up may differ from the characteristics of control group participants lost to follow-up. As a result, the intervention and control groups may no longer be exchangeable in the presence of loss to follow-up. Consequently, simple cross-sectional analyses conducted on the available data from followed-up participants in a clinical trial will generally not be adequate in trials with substantial loss-to follow-up. In contrast, longitudinal analyses conducted with linear mixed models may generate more robust estimates of effects of intervention because mixed longitudinal models generate unbiased estimates if data are missing at random conditional on covariates in the model, and it will often be the case that missingness depends on the values of outcomes measured prior to the loss to follow-up [20].

There are many alternatives to the use of mixed longitudinal models for analysing long-term follow-up data with substantial loss to follow-up. The literature on these methods is extensive [21]. One particularly useful approach is multiple imputation. Multiple imputation uses the available data (data from participants who were not lost to follow-up, as well as data obtained prior to loss to follow-up) to impute values for missing observations. The value of multiple imputation is that it can generate unbiased estimates with correct standard errors if the data are missing at random conditional on the available data. Helpful introductions to multiple imputation are provided in references [22–24].

### Truncation by death and other events

In clinical trials with long-term follow-up, particularly trials of elderly or very sick patients, substantial numbers of participants may die during the course of the trial. When a trial participant dies, any outcome that was to have been measured after the time of the participant’s death (other than whether the participant is dead or alive) is undefined. This does not present a problem for estimation of effects of intervention on all-cause mortality. However it does present a problem for estimation of effects of intervention on cause-specific mortality and non-mortality outcomes. If the intervention influences survival and there are characteristics of people that influence both survival and the outcome of interest then surviving participants in the intervention and control groups are not exchangeable. So a simple comparison of any outcome other than all-cause mortality between the intervention and control groups may generate estimates of effects of intervention that do not have a causal interpretation. This has been called the truncation by death problem [25].

As an illustration, consider a hypothetical trial where people with severe knee osteoarthritis are randomised to receive either knee surgery or a non-surgical control intervention. The outcome of interest in this trial is knee pain 12 months after randomisation. Suppose that treatment influences survival: people randomised to surgery have a higher risk of dying than people randomised to control. It is likely there would be some characteristics that influence both survival and pain (for example, patients in poor health might have a higher risk of death following surgery and higher levels of knee pain). If the comparison of 12 month knee pain outcomes was restricted to people who had survived to 12 months, it would be a comparison between non-exchangeable groups: the comparison would be between a control group in which participants with poor health and severe knee pain had been included and an intervention group in which participants with poor health and severe knee pain had been excluded by death. Again, the comparison of non-exchangeable groups could generate estimates that do not have a valid causal interpretation. Another way of saying this is that the analysis could generate estimates of the effect of intervention that are biased by conditioning on the post-randomisation variable of survival.

When there is truncation by death it may be tempting to use the methods described in the preceding section to impute observations for trial participants who died. But imputation of outcomes for participants who died is inadvisable because outcomes for participants who died are not missing, they are undefined. It usually does not make sense to think about what the outcomes would have been if the participant had not died.

The alternative is to calculate the survivor average causal effect (SACE) – the average effect of the intervention in participants who would have survived whether they were allocated to the intervention or control group. Unfortunately estimation of the SACE is more difficult than estimation of the CACE. When estimating the CACE we can assume that allocation had no effect on the outcomes of non-compliers, but when estimating the SACE we cannot make assumptions about outcomes of participants who died because their outcomes are undefined.

Nonetheless, we can estimate the SACE if we are prepared to make specific assumptions and hazard some guesses. One simple approach described by Chiba and Vanderweele [26] relies on the assumption that all participants who would survive in the control condition would also survive in the intervention condition (sometimes called the monotonicity assumption). Having made that assumption, an estimate of the SACE can be obtained by taking the intention to treat estimate from surviving trial participants and subtracting from it a guess of the difference between the average outcomes that would have been observed under intervention in people who would have survived with intervention and the average outcomes that would have been observed under intervention in people who would have survived without intervention. Vanderweele ([27], pp. 207–11) provides a short and clear description of this approach, as well as references to alternative approaches.

The preceding paragraphs considered how to estimate effects of intervention when the deaths of some trial participants cause trial outcomes to be undefined. A similar problem can arise when events other than death cause outcomes to be undefined. For example if, in a trial of an intervention for dental caries, some participants had a tooth extraction before the end of the study, any outcome measure that was subsequently to have been obtained from that tooth would be undefined. The methods described above for estimating effects of intervention in the presence of truncation by death can be used to estimate effects of intervention in the presence of truncation by other events as well as truncation by death.

## Conclusions

It is usually straightforward, with randomised trials, to obtain pragmatic estimates of the effect of intending to provide an intervention. But many clinical trialists and clinicians express an interest in the explanatory effect of intervention rather than in the pragmatic effect of intention to provide an intervention. The methods described in this paper provide methods that can be used to estimate the effects of intervention even in long-term trials with non-compliance, treatment switching, loss to follow-up and death.

The difficulties of conducting and analysing randomised trials with long term follow-up have caused many researchers to rely on observational studies for evidence of long-term effects. However, the shortcomings of using observational studies to infer causal effects of intervention are well known (see references [28, 29] for readable accounts). Using the methods described in this paper, it will often be possible to obtain estimates of the long-term effects of intervention from randomised trials even in the presence of non-compliance, treatment switching, loss to follow-up and death. Table 1 provides a summary of the issues, causal estimands, and statistical methods.

In the section on truncation by death and other events we considered issues that arise when observation of the outcome of interest is precluded by events such as death. The methods discussed in that section are quite new. However the closely related issue of censoring of time to event by the occurrence of an event that is not the outcome of primary interest has a long history in survival analysis, where it is referred to as the problem of competing risks. There are well-established methods for estimating the effects of intervention on the time to the event of interest in the presence of competing risks. The most widely used method is the method of competing risks regression described by Fine and Gray [27], which is an extension of the Cox proportional hazards model. The method of competing risks estimates quantities known as “sub-hazard ratios” instead of hazard ratios. The *causal* interpretation of hazard ratios and sub-hazard ratios is potentially problematic [30, 31].

Researchers who intend to conduct randomised trials with long-term follow-up should anticipate non-compliance, treatment switching, co-intervention, loss to follow-up and death, and design their trials in a way that facilitates the sorts of analyses described here. Specifically, at the planning stage, researchers should consider collecting data that is predictive of non-adherence, treatment switching and outcomes. Most of the statistical approaches described here rely on the availability of such data.

In the preceding discussion we separately described methods for estimating the effects of intervention from long-term trials with non-adherence, treatment switching, loss to follow-up and death. In many trials, more than one of these issues arises. Several authors have described applications combining two of the methods described here in the same analysis. For example, Dunn and colleagues describe estimation of effects of intervention in the presence of non-compliance and loss to follow-up [32], Toh and Hernan describe estimation of effects of intervention in the presence of treatment switching and loss to follow-up [19], and Daza and colleagues [33] describe estimation of effects of intervention in the presence of loss to follow-up and death. However, we caution that there has been little investigation of methods for or consequences of combining several of these methods simultaneously. That should be one of the focuses of research in this rapidly evolving field.

## Abbreviations

CACE: Complier average causal effect
SACE: Survivor average causal effect
